# Telitacicept for IgA nephropathy: a systematic review and meta-analysis of observational studies

**DOI:** 10.3389/fmed.2026.1894514

**Published:** 2026-08-04

**Authors:** Li Zheng, Xiaotong Gu, Chumeng Yang, Weina Zhang, Changhai Fu, Yan Wang, Xiaoyong Fang

**Affiliations:** 1 Department of Pharmacy, Beijing Genertec Aerospace Hospital, Beijing, China; 2 Department of Nephrology, Beijing Genertec Aerospace Hospital, Beijing, China; 3 Department of Cardiology, Beijing Hospital, National Center for Gerontology, National Clinical Research Center for Gerontology, The Key Laboratory of Geriatrics of NHC, Institute of Geriatric Medicine, Chinese Academy of Medical Sciences, Beijing, China; 4 Hospital Administration Office, Beijing Electric Power Hospital, Beijing, China

**Keywords:** IgA nephropathy, telitacicept, meta-analysis, 24-hour urinary protein quantity, estimated glomerular filtration rate, safety, GRADE

## Abstract

**Background:**

Telitacicept, a dual BAFF/APRIL inhibitor, is a potential targeted therapy for IgA nephropathy (IgAN), but its real-world efficacy and safety remain unclear.

**Methods:**

We systematically searched PubMed, Embase, Cochrane Central, ClinicalTrials.gov, CNKI, Wanfang, VIP, and SinoMed from inception to 2 March 2026 for observational studies of telitacicept in biopsy-confirmed IgAN. Eligible studies compared telitacicept monotherapy or telitacicept-based combination therapy with other immunosuppressive treatments. Outcomes included complete remission (CR), changes in 24-h urinary protein (Δ24hUPQ), estimated glomerular filtration rate (ΔeGFR), serum creatinine (ΔScr), albumin (ΔAlb), and adverse drug reactions (ADRs). Odds ratios (ORs) and mean differences (MDs) were pooled.

**Results:**

Eight observational studies involving 459 patients were included. Compared with other immunosuppressive therapies, telitacicept monotherapy showed no statistically significant differences in CR, Δ24hUPQ, ΔeGFR, ΔScr, and ΔAlb. Similarly, telitacicept-based combination therapy did not show statistically significant differences in CR, Δ24hUPQ, ΔeGFR, ΔScr, and ΔAlb. In terms of safety, telitacicept monotherapy was associated with a significantly lower incidence of ADRs than control treatments (OR 0.37, 95% CI 0.20–0.67). The certainty of evidence ranged from moderate to very low across outcomes.

**Conclusions:**

Current observational evidence suggests that telitacicept has a favorable safety profile and may offer clinical benefit in IgAN, particularly as a well-tolerated therapeutic option. Although superiority in efficacy outcomes was not statistically confirmed, the overall direction of effect tended to favor telitacicept in several analyses. Larger high-quality prospective studies are needed to further clarify its therapeutic value.

**Systematic review registration:**

The study was registered in the PROSPERO database (CRD420261296245), https://www.crd.york.ac.uk/PROSPERO/recorddashboard.

## 1 Introduction

IgA nephropathy (IgAN) is the most common primary glomerular disease worldwide and an important cause of chronic kidney disease and kidney failure (1, 2). Persistent proteinuria and declining kidney function are strongly associated with adverse renal outcomes (3, 4). Although optimized supportive therapy remains the foundation of treatment, many patients continue to progress, while the use of conventional immunosuppressive therapy is limited by variable efficacy and safety concerns (5–7). Therefore, effective and well-tolerated therapeutic options are still needed for patients with IgAN.

IgAN is characterized by immune dysregulation involving abnormal B-cell activation, increased production of galactose-deficient IgA1, and glomerular deposition of pathogenic immune complexes (8). B-cell activating factor (BAFF) and a proliferation-inducing ligand (APRIL) are key regulators of B-cell survival and antibody production and have been implicated in the pathogenesis of IgAN (9). Telitacicept, a dual BAFF/APRIL inhibitor, has therefore emerged as a potential targeted therapy for this disease (10). Recent clinical studies have suggested that telitacicept may improve disease remission in patients with IgAN, but the available evidence remains limited and has not been systematically evaluated.

In addition to renal injury, patients with IgAN often present with protein loss, chronic inflammation, and metabolic disturbances, all of which may influence overall clinical status during the course of chronic kidney disease (11–13). Among routinely measured laboratory markers, serum albumin is clinically relevant because it may reflect not only urinary protein loss but also systemic inflammatory burden and general physiological reserve (12). Therefore, evaluation of treatment response in IgAN should not be limited to renal endpoints alone, but may also include supportive biomarkers such as albumin to provide a more comprehensive assessment of disease status.

In this study, we conducted a systematic review and meta-analysis to evaluate the efficacy and safety of telitacicept in patients with IgAN. We also separately evaluated telitacicept monotherapy and telitacicept-based combination therapy to better define its potential role in the management of this disease. Because this review was designed to focus specifically on real-world evidence reflecting routine clinical practice, eligibility was restricted to observational studies. Randomized controlled trials (RCTs) were therefore not included in the pooled analysis, although their findings were considered in the interpretation of the evidence.

## 2 Methods

The study was performed following the Preferred Reporting Items for Systematic Reviews and Meta-Analyses (PRISMA) statement and registered in the PROSPERO database (CRD420261296245).

### 2.1 Search strategy and selection criteria

We searched PubMed, Embase, the Cochrane Central Register of Controlled Trials, the ClinicalTrials.gov, CNKI, Wanfang, VIP and SinoMed from database inception up to 2 March 2026, and screened the reference lists of relevant systematic reviews for additional trials. The search was conducted using a combination of controlled vocabulary (e.g., MeSH) and free-text terms. The search terms included “IgA Nephropathy,” “Telitacicept,” “RC18,” and “TACI-Fc”. The detailed search strategy of all databases can be seen in Supplementary Text S1.

Studies were included in this analysis if they met the following eligibility criteria: 1) The study design is observational, including cohort studies, case-control studies, and cross-sectional studies, which directly provide odds ratios (OR) or risk ratios (RR) along with their 95% confidence intervals (CI), or allow for these metrics to be calculated directly from the data presented in the article; 2) Patients with biopsy-confirmed IgAN who received telitacicept treatment; 3) Outcome Measures: Indicators that could be accurately measured and assessed in clinical studies and were applicable for evaluating changes in disease status following telitacicept treatment, such as the rate of complete remission (CR, which was defined as patients with 24-h proteinuria ≤ 0.3 g, ALB > 35 g/L and stable renal function (a decrease in eGFR ≤ 30%)), the change of 24-h urinary protein quantity (Δ24hUPQ), the change of estimated glomerular filtration rate (ΔeGFR), the change of serum creatinine (ΔScr), the change of albumin (ΔAlb), and adverse drug reactions (ADRs). We excluded animal studies, case, studies without clearly defined diagnostic criteria, and studies for which full text was unavailable.

### 2.2 Study selection and data extraction

All retrieved study records were imported into EndNote for management. Two authors (XTG and LZ) independently conducted an initial screening based on titles and abstracts, excluding studies that did not meet the inclusion criteria. The remaining articles were reviewed in full text to finalize their eligibility for inclusion in the analysis. Any disagreements between the two reviewers were resolved through discussion or, if necessary, by arbitration from a third author (YW).

Subsequently, two researchers (LZ and CMY) independently extracted data, including the study title, first author, publication year, study design, patient characteristics, interventions, treatment duration, and outcomes of interest. Discrepancies were resolved through discussion, with a third author (XYF) making the final decision when needed. For multiple reports of the same study (e.g., full-text articles, conference abstracts, and post-hoc analyses), all relevant data were consolidated and analyzed as a single study.

### 2.3 Risk of bias assessment

Two reviewers (LZ and WNZ) independently assessed all eligible studies. The risk of bias in non-randomized controlled trials was evaluated using the Risk Of Bias In Non-randomized Studies of Interventions (ROBINS-I) tool (14). Any discrepancies arising during the assessment process were resolved through discussion and consensus within the review team.

### 2.4 Grading the evidence

We assessed the certainty of evidence using the Grading of Recommendations Assessment, Development and Evaluation (GRADE) approach (15, 16). For each outcome and comparison, the certainty of evidence was rated as high, moderate, low, or very low by considering the domains of risk of bias, inconsistency, indirectness, imprecision, publication bias, intransitivity, and incoherence (17). Summary of findings tables were prepared in MAGICapp in accordance with GRADE methodology (18) (see detail in Supplementary Table S1).

### 2.5 Data analysis

Meta-analysis of the included studies was performed using RevMan 5.3 software provided by the Cochrane Collaboration. The OR with its 95%CI was selected as the effect size for dichotomous variables, and the mean difference (MD) with its 95% CI was selected for continuous variables. In cases of missing SDs, these were imputed following the guidelines of the Cochrane Handbook (19). The heterogeneity among studies was assessed using the chi-square test, and the degree of heterogeneity was quantitatively determined in combination with the *I*^2^ statistic. When *I*^2^ > 50%, heterogeneity among studies was considered present, and a random-effects model was adopted for unexplained heterogeneity.

Additionally, Stata software (version 18.0; StataCorp, College Station, TX, USA) was used to perform Egger's test and Begg's test to quantitatively assess publication bias among the included studies and to identify outliers (20).

## 3 Results

### 3.1 Search results and study characteristics

**Figure 1:**
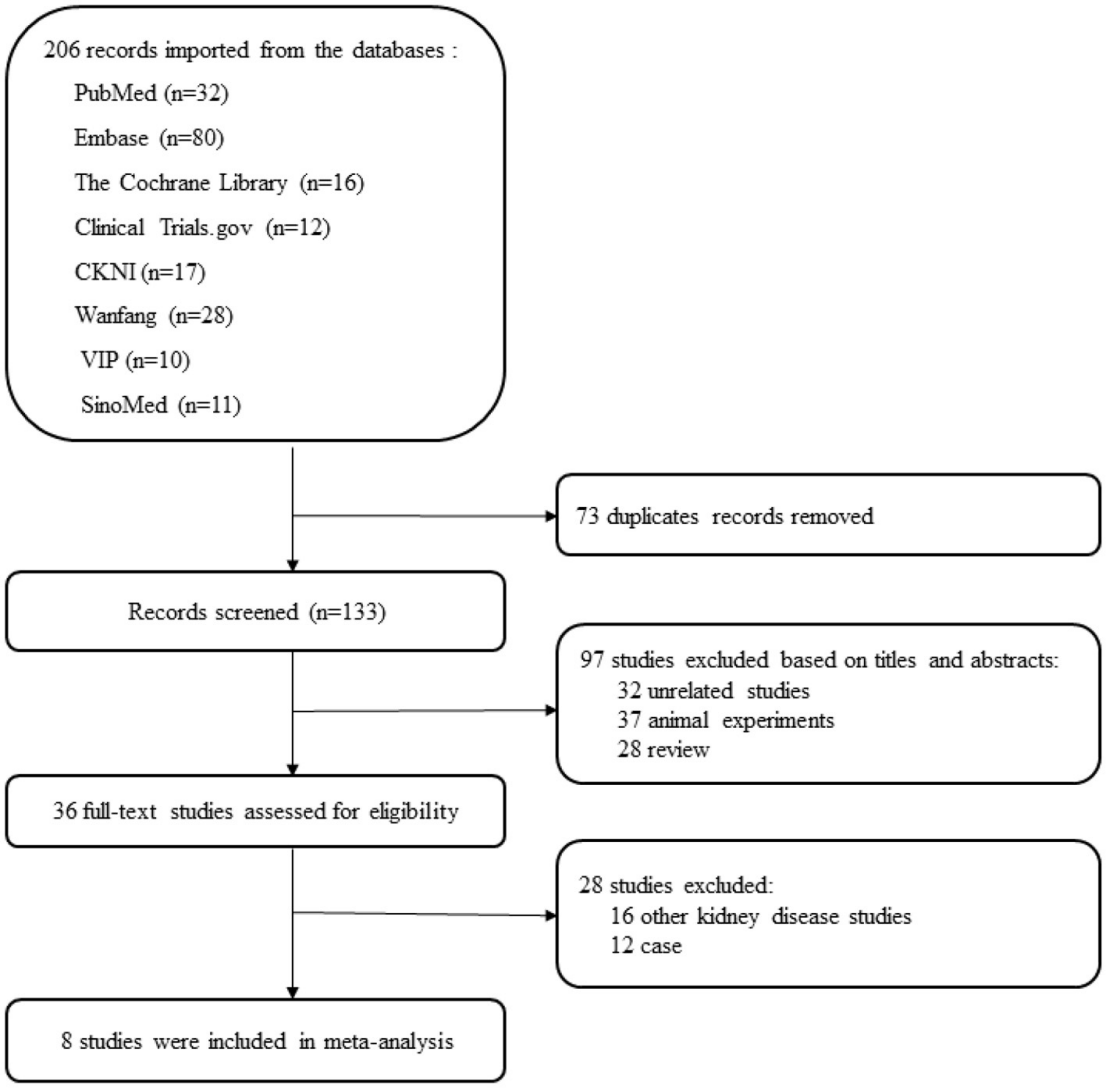
The flowchart of the study search.

A total of 206 records were identified through the electronic search, and 73 studies were removed after systematic duplication checks. After screening 133 titles and abstracts, 36 articles underwent full-text review. This process culminated in the final inclusion of 8 articles (Figure 1).

All included studies can be categorized into two distinct comparison indicators, namely telitacicept *vs*. other immunosuppressants and telitacicept combined with other immunosuppressants *vs*. other immunosuppressants alone. A total of 459 patients were enrolled, with a mean age ranging from 22 to 62 years, and treatment durations spanning from 3 to 12 months. The included studies were all published between 2024 and 2025. Table 1 lists the names of the other immunosuppressants and the detailed comparison measures of the included studies.

**Table 1:** The detailed characteristics of the included studies.

Comparison	References	Study type	Group	Mean age	No.	Male	Intervention	Duration (month)	Adjustment
Telitacicept vs. other immunosuppressants	Lingqiu Dong et al. (24)	Case–control study	Intervention	34.0	21	19	Telitacicept	3	Age, Sex, SBP, DBP, Hemoglobin, Albumin, Total triglyceride, Total cholesterol, Scr, eGFR, 24hUPQ, UPCR
			Control	31.0	21	17	Prednisone Acetate		
	Hui Li et al. (25)	Retrospective study	Intervention	35.91 ± 13.48	11	4	Telitacicept	6–12	Age, Sex, BMI, MAP, Oxford classification, Hypertension, Diabetes, Disease duration, Albumin, Scr, eGFR, 24hUPQ, RBC, IgA
			Control	38.50 ± 10.86	22	7	Prednisone Acetate		
	Meng Wang et al. (26)	Retrospective study	Intervention	38.15 ± 11.70	20	12	Telitacicept	6	Age, Sex, BMI, Hypertension, SBP, DBP, Diabetes, Treatment duration, Follow–up period, eGFR, Oxford classification, Albumin, Hemoglobin, 24hUPQ, URBC, Treatment
			Control	41.54 ± 12.80	28	12	Mycophenolate Mofetil		
	Yangyang He et al. (27)	Retrospective cohort study	Intervention	36.3 ± 10.8	48	18	Telitacicept	12	Age, Sex, BMI, Hypertension, SBP, DBP, BUN, UA, Hemoglobin, Scr, eGFR, Oxford classification, Albumin, 24hUPQ
			Control	37.4 ± 12.7	56	29	Mycophenolate Mofetil		
	Juntian Zhang et al. (28)	Retrospective cohort study	Intervention	36.1 ± 10.4	44	23	Telitacicept	6	Age, Sex, BMI, SBP, DBP, Scr, eGFR, UA, BUN, ALT, Total triglyceride, LDL–C, URBC, PLT, LYC, Albumin, 24hUPQ, Disease duration
			Control	37.6 ± 13.2	27	17	Mycophenolate Mofetil		
	Kun Yang et al. (21)	Retrospective cohort study	Intervention	41.30 ± 16.43	10	NA	Telitacicept	6	Age, Sex, BMI, Serum creatinine, eGFR, Hemoglobin, Albumin, 24hUPQ, TC, LDL–C, BUN, UA
			Control	45.50 ± 16.69	10	NA	Prednisone Acetate		
Telitacicept+ other immunosuppressants vs. other immunosuppressants	Fanqian Cheng et al. (22)	Retrospective cohort study	Intervention	38.27 ± 11.73	39	19	Telitacicept + Prednisone Acetate	12	Age, Sex, BMI, Hypertension, Diabetes, Scr, eGFR, Follow–up period, Hemoglobin, AST, ALT, BUN, TG, LDL–C, URBC, Albumin, 24hUPQ, Disease duration, Oxford classification
			Control	41.82 ± 12.04	38	20	Prednisone Acetate		
	Jing Luo et al. (23)	Retrospective cohort study	Intervention	37.25 ± 2.66	20	11	Telitacicept + Prednisone Acetate	12	Age, Sex, BMI, Diabetes, Scr, eGFR, Hemoglobin, ALT, Total triglyceride, LDL–C, Albumin, 24hUPQ.
			Control	40.41 ± 1.99	44	18	Prednisone Acetate		

BMI, body mass index; SBP, systolic blood pressure; DBP, diastolic blood pressure; UA, uric acid; BUN, blood urea nitrogen; ALT, alanine aminotransferase; AST, aspartate aminotransferase; LDL-C, low-density lipoprotein cholesterol; URBC, urinary red blood cell count; PLT, platelet count; LYC, lymphocyte count; TC, total cholesterol; TG, triglycerides; UPCR, urinary protein-to-creatinine ratio.

### 3.2 Risk of bias assessment

Table 2 presents the risk of bias assessment results for all included studies. Three of the studies (21–23) were rated as having a serious risk of bias, while the other five studies (24–28) were assessed as having a moderate risk of bias. One study (21) did not provide relevant information regarding the domain “Bias due to deviations from intended interventions”. Three studies (21–23) lacked information concerning “Bias due to missing data”. None of the included studies were found to have bias due to confounding, selection of participants, classification of interventions, measurement of outcomes, or selection of the reported result.

**Table 2:** The risk of bias assessment in included studies.

References	ROBINS–I (Non–Randomized Studies)							
	Bias due to confounding	Bias in selection of participants into the study	Bias in classification of interventions	Bias due to deviations from intended interventions	Bias due to missing data	Bias in measurement of outcomes	Bias in selection of the reported result	Overall bias
Lingqiu Dong et al. (24)	PN	PN	N	N	N	PN	N	Moderate
Hui Li et al. (25)	PN	PN	PN	N	N	PN	N	Moderate
Meng Wang et al. (26)	PN	PN	N	PN	PN	N	PN	Moderate
Yangyang He et al. (27)	PN	PN	N	N	N	N	PN	Moderate
Juntian Zhang et al. (28)	PN	PN	N	PN	PN	N	PN	Moderate
Kun Yang et al. (21)	PN	PN	PN	NI	NI	N	PN	Serious
Fanqian Cheng et al. (22)	PN	PN	N	PN	NI	N	PN	Serious
Jing Luo et al. (23)	PN	PN	PN	PN	NI	N	PN	Serious

### 3.3 Meta-analysis

#### 3.3.1 Effect of telitacicept in IgAN patients

**Figure 2:**
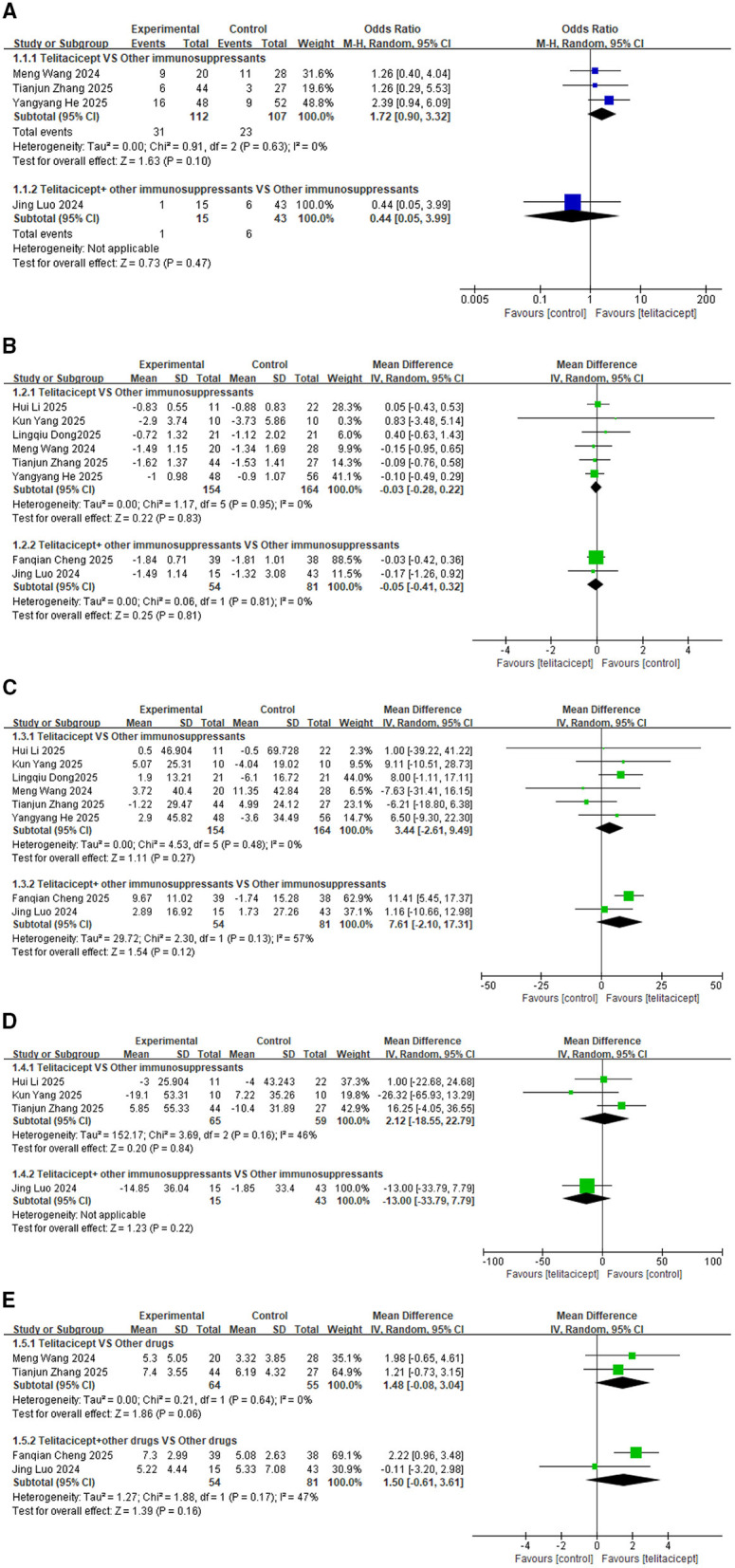
**(A)** Meta-analysis results on the incidence of CR. **(B)** Meta-analysis results on mean reduction in 24hUPQ. **(C)** Meta-analysis results on mean increase in eGFR. **(D)** Meta-analysis results on mean reduction in Scr. **(E)** Meta-analysis results on mean increase in Alb.

Four studies (23, 26–28)reported the CR of telitacicept as monotherapy or in combination with other immunosuppressants in patients with IgAN. Among these, three studies (26–28) evaluated the efficacy of telitacicept monotherapy, and one study (23) evaluated the efficacy of telitacicept combination with other immunosuppressants. The meta-analysis results showed that, for both monotherapy (OR: 1.72, 95%CI: 0.90–3.32, *P* = 0.10, the certainty of evidence was moderate) and combination therapy (OR: 0.44, 95%CI: 0.05–3.99, the certainty of evidence was very low), telitacicept showed no statistically significant difference in increasing CR compared to other treatments (Figure 2a).

Eight studies (21–28) assessed the effect of telitacicept on Δ24hUPQ. Ultimately, six studies (21, 24–28) reported the effect of telitacicept on Δ24hUPQ, and two studies (22, 23) reported the effect of combination therapy. Meta-analysis revealed that, for both monotherapy (MD:−0.03, 95%CI:−0.28 to 0.22, *P* = 0.83, the certainty of evidence was moderate) and combination therapy (MD:−0.05, 95%CI:−0.41 to 0.32, *P* = 0.81, the certainty of evidence was moderate), telitacicept showed no statistically significant difference in reducing 24 hUPQ compared to other treatments. However, the weight of evidence tended to favor the therapeutic benefit of telitacicept (Figure 2b).

Eight studies (21–28) reported the effect of telitacicept on ΔeGFR. Among these, six studies (21, 24–28) assessed the effect of telitacicept monotherapy. The results showed that, for both monotherapy (MD: 3.44, 95%CI:−2.61 to 9.49, *P* = 0.27, the certainty of evidence was moderate) and combination therapy (MD: 7.61, 95%CI:−2.10 to 17.31, *P* = 0.12, the certainty of evidence was moderate), telitacicept showed no statistically significant difference in increasing eGFR levels compared to other treatments (Figure 2c). However, the weight of evidence tended to favor the therapeutic benefit of telitacicept.

A total of four studies (21, 23, 25, 28) reported the changes in Scr levels. Three studies (21, 25, 28) reported the efficacy of telitacicept monotherapy, while one study (23) reported the efficacy of combination therapy with other immunosuppressants. The meta-analysis results showed whether comparing telitacicept *vs*. other immunosuppressants (MD: 2.12, 95%CI:−18.55 to 22.79, *P* = 0.84, the certainty of evidence was very low) or telitacicept combined with other immunosuppressants *vs*. other immunosuppressants (MD:−13.00, 95%CI:−33.79 to 7.79, *P* = 0.22, the certainty of evidence was very low), the differences in ΔScr were not statistically significant (Figure 2d).

Four studies (22, 23, 26, 28) reported the changes in Alb, with two studies (26, 28) reporting the efficacy of monotherapy with telitacicept and two studies (22, 23) reporting the efficacy of combination therapy with other immunosuppressants. The meta-analysis results showed that there was no significant statistical difference between monotherapy and other immunosuppressants (MD: 1.48, 95%CI:−0.08 to 3.04, *P* = 0.06, the certainty of evidence was very low) or combination therapy with other immunosuppressants compared to other immunosuppressants (MD: 1.50, 95%CI:−0.61 to 3.61, *P* = 0.16, the certainty of evidence was very low). But the weight of evidence tended to favor the therapeutic benefit of telitacicept (Figure 2e).

#### 3.3.2 Safety of telitacicept on the outcomes in IgAN patients

**Figure 3:**
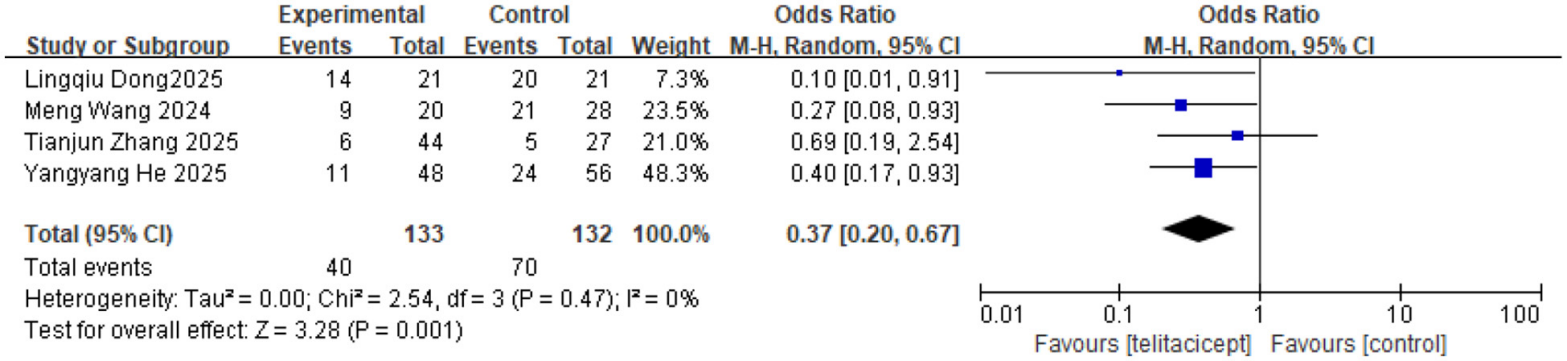
Meta-analysis results on the incidence of ADRs.

ADRs were reported in four studies (24, 26–28), all of which compared telitacicept monotherapy with other immunosuppressants. The results showed that the incidence of ADRs in the telitacicept group was significantly lower than that in the other immunosuppressants group (OR: 0.37, 95%CI: 0.20–0.67, *P* = 0.001, the certainty of evidence was moderate) (Figure 3).

### 3.4 Sensitivity analysis and Publication bias

We conducted a sensitivity analysis to assess the stability and reliability of the meta-analysis results. By sequentially excluding each of the eight studies (21–28), the results showed that no individual study significantly altered the therapeutic efficacy of telitacicept, indicating that the findings of the meta-analysis are robust (see Supplementary Figures S1-S6).

Because the number of eligible studies for each outcome was limited, formal assessment of publication bias was not performed.

## 4 Discussion

In this meta-analysis, eight studies involving 459 patients with IgAN were included to evaluate the efficacy and safety of telitacicept. According to treatment strategy, the eligible studies were categorized into telitacicept monotherapy versus other immunosuppressants and telitacicept-based combination therapy versus other immunosuppressants alone. The pooled results showed that telitacicept monotherapy was not associated with statistically significant differences in CR, Δ24 hUPQ, ΔeGFR, ΔScr or ΔAlb compared with other immunosuppressive therapies. Similarly, telitacicept-based combination therapy also did not show significant differences in CR, Δ24 hUPQ, ΔeGFR, ΔScr or ΔAlb. In terms of safety, telitacicept monotherapy was associated with a significantly lower incidence of adverse drug reactions (ADRs) than the control group. Sensitivity analyses supported the stability of the main finding.

One of the findings of the present study was that telitacicept-based combination therapy showed a potential signal toward improved complete remission (CR), but the between-group difference did not reach statistical significance in the pooled analysis. Therefore, the current evidence does not support a firm conclusion that combination therapy is superior to conventional immunosuppressive treatment in achieving CR in patients with IgAN. Even so, this trend may still be clinically relevant and deserves further investigation in larger and better-designed studies. CR remains a meaningful endpoint in IgAN because it reflects an integrated treatment response, including reduction of proteinuria, preservation of renal function, and improvement in overall disease control. The possible benefit of telitacicept in the combination setting may be related to its dual inhibition of BAFF and APRIL, which can modulate B-cell activation, plasma cell survival, and the production of pathogenic IgA-containing immune complexes. Given the central role of abnormal IgA-mediated immune responses in the pathogenesis of IgAN, this mechanism provides a biologically plausible rationale for the use of telitacicept as an adjunctive therapeutic option, although its effect on remission still requires confirmation (29–31).

By contrast, telitacicept monotherapy did not show a statistically significant advantage in CR, and neither monotherapy nor combination therapy demonstrated significant superiority in 24hUPQ. These findings should be interpreted cautiously rather than viewed as evidence of no therapeutic effect. CR is a relatively stringent endpoint that depends on multiple factors, including the degree of proteinuria reduction, preservation of renal function, and the timing of evaluation (3, 5). Similarly, 24hUPQ is influenced by baseline proteinuria burden, concomitant renin-angiotensin system blockade, blood pressure control, treatment duration, and biological variability (3, 5, 32). In the included studies, background therapies were not uniform, and comparator regimens varied across cohorts, which likely reduced the ability to detect between-group differences. In addition, IgAN is a chronic disease with slow progression, and meaningful changes in proteinuria or remission status may require a longer observation period than was available in most of the included studies (3, 5). Therefore, the absence of statistical significance for 24 hUPQ, and for CR in the monotherapy subgroup, should be interpreted as insufficient evidence of benefit at present rather than evidence against a therapeutic effect.

The findings for kidney function also deserve careful interpretation. Neither monotherapy nor combination therapy showed a statistically significant difference in eGFR compared with control treatment in the pooled analysis, although the direction of effect generally favored telitacicept. Similarly, no significant difference was observed for Scr. These findings suggest that current evidence is insufficient to confirm a definite renal functional benefit, but they do not exclude the possibility of a modest effect that could not be detected because of limited sample size and short follow-up. eGFR remains a clinically important endpoint in IgAN because long-term prognosis is determined largely by the progressive loss of filtration capacity (3, 5, 33). However, in relatively small and mostly observational studies, short-term changes in eGFR may be subtle and difficult to detect statistically. Scr is also affected by muscle mass, hydration status, and hemodynamic variation, and therefore may be less sensitive to modest changes in kidney function (33). Accordingly, the current pooled results should be interpreted as showing no statistically significant renal functional advantage of telitacicept at present, although the favorable trend in eGFR may warrant further investigation.

The findings for Alb also require cautious interpretation. No statistically significant difference in Alb was observed between telitacicept and control treatment in the pooled analysis, although the direction of effect tended to favor telitacicept. Alb is a complex indicator that reflects not only urinary protein loss but also inflammatory burden, nutritional status, hepatic synthesis, and fluid balance (13, 34). Consequently, the lack of a significant difference in Alb does not necessarily contradict a potential treatment benefit, but it does indicate that albumin is unlikely to be a sufficiently sensitive standalone marker for evaluating the short-term efficacy of telitacicept in the currently available studies.

Safety is another important consideration. The pooled safety outcome in this study referred to overall drug-related adverse reactions as reported in the original studies. Because the included studies did not provide sufficiently consistent data on specific event categories or severity grading, further stratified analyses were not feasible. The present analysis showed that telitacicept monotherapy was associated with a lower incidence of ADRs than control treatment. This is clinically relevant because conventional immunosuppressive regimens used in IgAN, such as glucocorticoids and mycophenolate mofetil, are often limited by infection risk, metabolic disturbances, gastrointestinal symptoms, liver dysfunction, and hematologic toxicity (7, 35). A treatment that offers comparable efficacy with fewer ADRs may therefore have practical value, particularly for patients requiring prolonged therapy or those at higher risk of treatment-related complications. However, because the available evidence on ADRs was limited, especially for combination therapy, the comparative safety of telitacicept-based combination regimens remains uncertain.

The current findings are broadly consistent with the emerging mechanistic understanding of BAFF/APRIL-targeted therapy in IgAN. Telitacicept inhibits both BAFF and APRIL, thereby suppressing B-cell activation and plasma cell survival and potentially reducing the production of pathogenic IgA and related immune complexes (29–31). This mechanism provides a biologically plausible rationale for its therapeutic role in IgAN (29–31, 36). However, although the present analysis showed a significant benefit for CR in the combination-therapy setting, it did not demonstrate statistically significant superiority for proteinuria-related outcomes or kidney function indices. Therefore, the available evidence supports potential clinical benefit, but remains insufficient to define the full therapeutic value of telitacicept.

It should also be noted that randomized trial evidence on telitacicept in IgAN is already available. In particular, the phase II study (11) and III study (37) reported beneficial effects of telitacicept on proteinuria reduction in patients with IgAN, supporting its potential as a targeted therapy. Therefore, the RCTs provide important efficacy evidence under controlled conditions, but it does not directly address the comparative clinical question examined in many real-world studies. For this reason, the present review focused specifically on observational evidence to better reflect real-world clinical application. Nevertheless, the exclusion of RCTs means that the certainty and completeness of the overall evidence base remain limited, and future systematic reviews may benefit from analyzing RCTs and observational studies separately.

The present study has several strengths. First, it systematically synthesized the available evidence on telitacicept in IgAN while distinguishing between monotherapy and combination therapy, thereby providing a more nuanced assessment of treatment effects. Second, the analysis considered not only efficacy outcomes but also adverse events, which are highly relevant for clinical decision-making. Third, the use of sensitivity analyses and publication bias assessment increased confidence in the stability of the main findings. These strengths support the clinical relevance of the current analysis despite the limited size of the evidence base.

Several limitations of this meta-analysis should be acknowledged. First, the number of included studies was relatively small, and most were retrospective observational studies, which may limit the strength of causal inference. Second, although continuous outcomes were pooled using change-from-baseline values, these measures may still have been influenced by differences across studies in baseline disease severity, blood pressure control, concomitant use of RAS blockade or steroids, and follow-up duration. Third, the comparator treatments were clinically heterogeneous, including prednisone acetate, mycophenolate mofetil, and prednisone-based regimens, and some comparisons involved telitacicept plus prednisone versus prednisone alone. Pooling these regimens into a single comparator category may have obscured clinically meaningful differences. We carefully considered performing subgroup analyses according to comparator type. However, because the number of included studies was limited and each comparator subgroup contained too few studies, such analyses would have been statistically underpowered and potentially unstable. Fourth, randomized controlled trials were not included in the quantitative synthesis, as this review was designed to focus on real-world observational evidence; therefore, the overall certainty and completeness of the evidence remain limited. Finally, the limitation is the relatively short follow-up duration of the included studies, which ranged from 3 to 12 months. For IgA nephropathy, clinically meaningful outcomes include sustained proteinuria remission, annual eGFR slope, substantial decline in kidney function, and progression to kidney failure/ESKD. Therefore, the outcomes assessed in the present meta-analysis mainly reflect short-term clinical and biochemical responses rather than long-term renal protection. Accordingly, the findings should be interpreted as evidence of short-term efficacy and safety signals only, and the effect of telitacicept on long-term renal prognosis remains uncertain. Future studies with longer follow-up are needed to clarify its impact on renal survival and disease progression.

Overall, telitacicept appears to have promising clinical potential in the treatment of IgAN, particularly as an adjunctive therapy for improving complete remission and as a monotherapy option with favorable tolerability. However, the current pooled evidence did not demonstrate statistically significant superiority for 24 hUPQ, eGFR, Scr, or Alb. As treatment paradigms for IgAN continue to evolve toward mechanism-based and individualized approaches, BAFF/APRIL-targeted therapy may become an important therapeutic option (29–31, 36). Nevertheless, further multicenter randomized controlled trials with larger sample sizes, longer follow-up, and standardized outcome definitions are needed to confirm these findings. Future studies should also incorporate nutritional indicators and inflammatory biomarkers to more fully evaluate the clinical value of telitacicept in the comprehensive management of IgAN.

## 5 Conclusion

In this systematic review and meta-analysis of observational studies, telitacicept did not show statistically significant superiority over other immunosuppressive therapies in complete remission, proteinuria, renal function, serum creatinine, or albumin outcomes in patients with IgA nephropathy, whether used as monotherapy or in combination therapy. Telitacicept monotherapy was associated with a lower incidence of drug-related adverse reactions, suggesting a potential safety advantage. However, given the limited sample size, observational nature of the included studies, and the low certainty of evidence for several outcomes, these findings should be interpreted with caution. Further large-scale, high-quality prospective studies are needed to confirm the efficacy and safety of telitacicept in IgAN.

## Data Availability

The original contributions presented in the study are included in the article/supplementary material, further inquiries can be directed to the corresponding authors.
