# Supplementary material for: Telitacicept for IgA nephropathy: a systematic review and meta-analysis of observational studies
Source: Front Med (Lausanne). 2026 Aug 4;13:1894514. doi: 10.3389/fmed.2026.1894514 (PMC13487682; doi:10.3389/fmed.2026.1894514)
Supplement: Supplementary file 1 [file Table_1.docx]

| **Section and Topic** | **Item #** | **Checklist item** | **Location where item is reported** |
| --- | --- | --- | --- |
| **TITLE** | | |  |
| Title | 1 | Identify the report as a systematic review. | Title page: "Telitacicept for IgA Nephropathy: Evidence from a Systematic Review and Meta-Analysis" |
| **ABSTRACT** | | |  |
| Abstract | 2 | See the PRISMA 2020 for Abstracts checklist. | Abstract section (structured abstract includes Background, Methods, Results, Conclusions, Keywords) |
| **INTRODUCTION** | | |  |
| Rationale | 3 | Describe the rationale for the review in the context of existing knowledge. | Section 1 (Introduction), paragraphs 1-3 |
| Objectives | 4 | Provide an explicit statement of the objective(s) or question(s) the review addresses. | Section 1, last paragraph: "Therefore, we conducted a systematic review and meta-analysis to evaluate the efficacy and safety of telitacicept in patients with IgAN..." |
| **METHODS** | | |  |
| Eligibility criteria | 5 | Specify the inclusion and exclusion criteria for the review and how studies were grouped for the syntheses. | Section 2.1 (Search Strategy and Selection Criteria): eligibility criteria (study design, patients, outcomes) and grouping into monotherapy vs combination therapy |
| Information sources | 6 | Specify all databases, registers, websites, organisations, reference lists and other sources searched or consulted to identify studies. Specify the date when each source was last searched or consulted. | Section 2.1: "PubMed, Embase, the Cochrane Central Register of Controlled Trials, ClinicalTrials.gov, CNKI, Wanfang, VIP and SinoMed from database inception up to 2 March 2026" |
| Search strategy | 7 | Present the full search strategies for all databases, registers and websites, including any filters and limits used. | Section 2.1: search terms provided; detailed strategy in Supplementary Text S1 |
| Selection process | 8 | Specify the methods used to decide whether a study met the inclusion criteria of the review, including how many reviewers screened each record and each report retrieved, whether they worked independently, and if applicable, details of automation tools used in the process. | Section 2.2: two authors (XTG and LZ) independently screened titles/abstracts; disagreements resolved by discussion or third author (YW); EndNote used for management |
| Data collection process | 9 | Specify the methods used to collect data from reports, including how many reviewers collected data from each report, whether they worked independently, any processes for obtaining or confirming data from study investigators, and if applicable, details of automation tools used in the process. | Section 2.2: two researchers (LZ and CMY) independently extracted data; discrepancies resolved by discussion, third author (YTG) if needed |
| Data items | 10a | List and define all outcomes for which data were sought. Specify whether all results that were compatible with each outcome domain in each study were sought (e.g. for all measures, time points, analyses), and if not, the methods used to decide which results to collect. | Section 2.1: outcomes defined (CR, 24hUPQ, eGFR, Scr, Alb, ADRs) with definitions (e.g., CR defined as 24hUPQ ≤0.3 g, ALB >35 g/L, eGFR decrease ≤30%) |
|  | 10b | List and define all other variables for which data were sought (e.g. participant and intervention characteristics, funding sources). Describe any assumptions made about any missing or unclear information. | Section 2.2 and Table 1: patient characteristics, interventions, treatment duration; Section 2.5: missing SDs imputed following Cochrane Handbook |
| Study risk of bias assessment | 11 | Specify the methods used to assess risk of bias in the included studies, including details of the tool(s) used, how many reviewers assessed each study and whether they worked independently, and if applicable, details of automation tools used in the process. | Section 2.3: ROBINS-I tool; two reviewers (LZ and WNZ) independently assessed; disagreements resolved by discussion |
| Effect measures | 12 | Specify for each outcome the effect measure(s) (e.g. risk ratio, mean difference) used in the synthesis or presentation of results. | Section 2.5: RR for dichotomous variables, MD for continuous variables; OR reported for ADRs and CR in results |
| Synthesis methods | 13a | Describe the processes used to decide which studies were eligible for each synthesis (e.g. tabulating the study intervention characteristics and comparing against the planned groups for each synthesis (item #5)). | Section 3.1 and Table 1: studies categorized into telitacicept vs other drugs and telitacicept+other drugs vs other drugs |
|  | 13b | Describe any methods required to prepare the data for presentation or synthesis, such as handling of missing summary statistics, or data conversions. | Section 2.5: missing SDs imputed per Cochrane Handbook |
|  | 13c | Describe any methods used to tabulate or visually display results of individual studies and syntheses. | Figures 2a-2e, Figure 3; Table 1, Table 2, Table 3 |
|  | 13d | Describe any methods used to synthesize results and provide a rationale for the choice(s). If meta-analysis was performed, describe the model(s), method(s) to identify the presence and extent of statistical heterogeneity, and software package(s) used. | Section 2.5: RevMan 5.3; chi-square test and I² statistic; random-effects model if I²>50% |
|  | 13e | Describe any methods used to explore possible causes of heterogeneity among study results (e.g. subgroup analysis, meta-regression). | Subgroup analysis by monotherapy vs combination therapy (pre-specified in Methods and presented in Results) |
|  | 13f | Describe any sensitivity analyses conducted to assess robustness of the synthesized results. | Section 3.4: sensitivity analysis by sequentially excluding each study; results in Supplementary Figures S1-S6 |
| Reporting bias assessment | 14 | Describe any methods used to assess risk of bias due to missing results in a synthesis (arising from reporting biases). | Section 2.5: Egger's test and Begg's test for publication bias |
| Certainty assessment | 15 | Describe any methods used to assess certainty (or confidence) in the body of evidence for an outcome. | Section 2.4: GRADE approach; summary of findings in Supplementary Table S1 |
| **RESULTS** | | |  |
| Study selection | 16a | Describe the results of the search and selection process, from the number of records identified in the search to the number of studies included in the review, ideally using a flow diagram. | Section 3.1: "206 records identified... 73 removed... 133 screened... 36 full-text reviewed... 10 articles included"; Figure 1 flow diagram |
|  | 16b | Cite studies that might appear to meet the inclusion criteria, but which were excluded, and explain why they were excluded. | Not explicitly listed; exclusion reasons implied by screening process (e.g., animal studies, case reports, no full text) - see Section 2.1 |
| Study characteristics | 17 | Cite each included study and present its characteristics. | Table 1: lists all 10 included studies (Dong 2025, Li H 2025, Weng 2025, Wang 2025, He 2025, Zhang 2025, Li S 2025, Yang 2025, Cheng 2025, Luo 2025) with characteristics |
| Risk of bias in studies | 18 | Present assessments of risk of bias for each included study. | Table 2: ROBINS-I assessment for each study |
| Results of individual studies | 19 | For all outcomes, present, for each study: (a) summary statistics for each group (where appropriate) and (b) an effect estimate and its precision (e.g. confidence/credible interval), ideally using structured tables or plots. | Results presented as forest plots (Figures 2a-2e, Figure 3) and summarized in text; individual study data not shown in tables but meta-analysis aggregates are provided |
| Results of syntheses | 20a | For each synthesis, briefly summarise the characteristics and risk of bias among contributing studies. | Section 3.3: each outcome includes certainty of evidence (e.g., moderate, low, very low) referencing GRADE; risk of bias in Table 2 |
|  | 20b | Present results of all statistical syntheses conducted. If meta-analysis was done, present for each the summary estimate and its precision (e.g. confidence/credible interval) and measures of statistical heterogeneity. If comparing groups, describe the direction of the effect. | Figures 2a-2e and Figure 3: each shows OR/MD, 95% CI, P-value, I², and model used |
|  | 20c | Present results of all investigations of possible causes of heterogeneity among study results. | Subgroup analyses (monotherapy vs combination) performed; heterogeneity reported via I²; no meta-regression |
|  | 20d | Present results of all sensitivity analyses conducted to assess the robustness of the synthesized results. | Section 3.4: "no individual study significantly altered the therapeutic efficacy" – detailed in Supplementary Figures S1-S6 |
| Reporting biases | 21 | Present assessments of risk of bias due to missing results (arising from reporting biases) for each synthesis assessed. | Section 3.5 and Table 3: Egger's test P-values >0.05 for all outcomes, indicating no evidence of publication bias |
| Certainty of evidence | 22 | Present assessments of certainty (or confidence) in the body of evidence for each outcome assessed. | Section 3.3: certainty reported for each outcome (e.g., "moderate-certainty evidence" for eGFR, "very low" for Scr and Alb) |
| **DISCUSSION** | | |  |
| Discussion | 23a | Provide a general interpretation of the results in the context of other evidence. | Section 4, paragraphs 1-2 and 5-6 |
|  | 23b | Discuss any limitations of the evidence included in the review. | Section 4, paragraphs 8-9: limitations (observational studies, small sample size, short follow-up, heterogeneity, risk of bias) |
|  | 23c | Discuss any limitations of the review processes used. | Section 4, paragraph 9: acknowledges limitations of review (small number of studies, limited power for publication bias tests, etc.) |
|  | 23d | Discuss implications of the results for practice, policy, and future research. | Section 4, paragraphs 10-11: need for multicenter RCTs, biomarker studies, precision medicine |
| **OTHER INFORMATION** | | |  |
| Registration and protocol | 24a | Provide registration information for the review, including register name and registration number, or state that the review was not registered. | Section 2: "registered in the PROSPERO database (CRD420261296245)" |
|  | 24b | Indicate where the review protocol can be accessed, or state that a protocol was not prepared. | PROSPERO registration; protocol not separately indicated – implied available via PROSPERO |
|  | 24c | Describe and explain any amendments to information provided at registration or in the protocol. | No amendments reported |
| Support | 25 | Describe sources of financial or non-financial support for the review, and the role of the funders or sponsors in the review. | Not reported in manuscript; "Conflict of interest" states no competing interests, but no funding statement |
| Competing interests | 26 | Declare any competing interests of review authors. | "Conflict of interest: The authors declare no competing interests." |
| Availability of data, code and other materials | 27 | Report which of the following are publicly available and where they can be found: template data collection forms; data extracted from included studies; data used for all analyses; analytic code; any other materials used in the review. | "Data availability statement: The data supporting the findings of this study are included in this article and its supplementary materials. Further inquiries can be directed to the corresponding author upon reasonable request." |

*From:*  Page MJ, McKenzie JE, Bossuyt PM, Boutron I, Hoffmann TC, Mulrow CD, et al. The PRISMA 2020 statement: an updated guideline for reporting systematic reviews. BMJ 2021;372:n71. doi: 10.1136/bmj.n71. This work is licensed under CC BY 4.0. To view a copy of this license, visit <https://creativecommons.org/licenses/by/4.0/>
