# Supplementary material for: Telitacicept for IgA nephropathy: a systematic review and meta-analysis of observational studies
Source: Front Med (Lausanne). 2026 Aug 4;13:1894514. doi: 10.3389/fmed.2026.1894514 (PMC13487682; doi:10.3389/fmed.2026.1894514)
Supplement: Supplementary file 2 [file Table_2.docx]

**Text S1**

**1) CNKI**

#1 ((SU='肾炎' AND SU='IgA') OR (SU='IgA 肾病' OR SU='IgA 肾脏病' OR SU='Berger病' OR SU='IgA 肾炎' OR SU='IgAN'))

#2 SU='泰它西普' OR TI='泰它西普'

#3 #1 AND #2

**2) WanFang**

#1 ((主题: 肾炎 AND 主题: IgA) OR (主题: IgA 肾病 OR 主题: IgA 肾脏病 OR 主题: Berger病 OR 主题: IgA 肾炎 OR 主题: IgAN))

#2 主题: 泰它西普 OR 题名或关键词: 泰它西普

#3 #1 AND #2

**3) VIP**

#1 ((M=肾炎 AND M=IgA) OR (M=IgA 肾病 OR M=IgA 肾脏病 OR M=Berger病 OR M=IgA 肾炎 OR M=IgAN))

#2 M=泰它西普

#3 1 AND #2

**4) SinoMed**

#1 "IgA 肾病"[不加权:扩展] OR "IgA 肾脏病"[不加权:扩展] OR "Berger病"[不加权:扩展] OR "IgA 肾炎"[不加权:扩展] OR "IgAN"[不加权:扩展]

#2 “泰它西普”[常用字段] OR “RC18”[常用字段] OR “TACI-Fc融合蛋白”[常用字段]

#3 #1 AND #2

**5) PubMed**

#1 "Telitacicept"[Title/Abstract] OR "RC18"[Title/Abstract] OR "TACI-Fc"[Title/Abstract]

#2 "IgA Nephropathy"[Mesh] OR "Glomerulonephritis, IGA"[Mesh]

#3 "Berger‘s Disease"[Title/Abstract] OR "Immunoglobulin A Nephropathy"[Title/Abstract] OR "IgAN"[Title/Abstract]

#4 #2 OR #3

#5 #1 AND #4

**6) Embase**

#1 ‘telitacicept‘/exp OR ‘telitacicept‘:ti,ab,kw OR ‘rc18‘:ti,ab,kw OR ‘taci-fc‘:ti,ab,kw

#2 ‘iga nephropathy‘/exp

#3 ‘berger disease‘:ti,ab,kw OR ‘immunoglobulin a nephropathy‘:ti,ab,kw OR ‘igan‘:ti,ab,kw

#4 #2 OR #3

#5 #1 AND #4

**7) The Cochrane Library**

#1 (iga nephropathy):ti,ab,kw OR (Immunoglobulin a Nephropathy):ti,ab,kw OR (immunoglobulin a nephropathy):ti,ab,kw OR (iga glomeruloneph):ti,ab,kw OR (berger disease):ti,ab,kw OR (IgAGN):ti,ab,kw OR (igAN):ti,ab,kw

#2 (Telitacicept):ti,ab,kw OR (RC18):ti,ab,kw OR (TACI-Fc):ti,ab,kw

#9 #1 AND #2

**8) Clinical Trials**

Intervention/treatment: Telitacicept OR RC18 OR TACI-Fc

Condition/disease: iga nephropathy OR Immunoglobulin a Nephropathy OR immunoglobulin a nephropathy OR iga glomeruloneph OR berger disease OR IgAGN OR IgaN

**Table S1**

Population: IgAN patients

Intervention: Telitacicept OR Telitacicept+ other immunosuppressants

Comparator: other immunosuppressants

**Table S1 Summary of findings of GRADE assessment**

| **Outcome**  Timeframe | **Study results and measurements** | **Absolute effect estimates** | | **Certainty of the evidence**  (Quality of evidence) | **Summary** |
| --- | --- | --- | --- | --- | --- |
|  |  | IgaN | Telitacicept |  |  |
| CR - Telitacicept VS Other immunosuppressants | Odds ratio: 1.25  (CI 95% 0.49 - 3.2)  Based on data from 219 participants in 3 studies^1^ | **298**  per 1000 | **346**  per 1000 | **Moderate**  Due to serious risk of bias, Due to serious imprecision^2^ | Compared with Other drugs, telitacicept may not increase the rate of CR. |
|  |  | Difference: **49 more per 1000**  (CI 95% 126 fewer - 278 more) | |  |  |
| CR - Telitacicept+ other immunosuppressants VS Other immunosuppressants | Odds ratio: 0.44  (CI 95% 0.05 – 3.99)  Based on data from 58 participants in 1 studies^3^ | **298**  per 1000 | **157**  per 1000 | **Very low**  Due to serious risk of bias, Due to serious imprecision, Due to serious inconsistency^4^ | Compared with Other drugs, telitacicept+ other drugs may not increase the rate of CR. |
|  |  | Difference: **141 fewer per 1000**  (CI 95% 277fewer - 331 fewer) | |  |  |
| ADR | Odds ratio: 0.37  (CI 95% 0.2 - 0.67)  Based on data from 265 participants in 4 studies^5^ | **530**  per 1000 | **294**  per 1000 | **Moderate**  Due to serious risk of bias, Due to serious imprecision^6^ | Compared with other immunosuppressants, telitacicept may reduce the rate of ADR. |
|  |  | Difference: **236 fewer per 1000**  (CI 95% 346 fewer - 100 fewer) | |  |  |
| 24h-UTP - Telitacicept VS Other immunosuppressants | Measured by:  Scale: -  Based on data from 318 participants in 6 studies^7^ |  |  | **Moderate**  Due to very serious risk of bias, Due to serious risk of bias^8^ | Compared with other immunosuppressants, telitacicept may not reduce 24hUTP. |
|  |  | Difference: **MD 0.03 lower**  (CI 95% 0.28 lower - 0.23 higher) | |  |  |
| 24h-UTP - Telitacicept+ other immunosuppressants VS Other immunosuppressants | Measured by:  Scale: -  Based on data from 135 participants in 2 studies^9^ |  |  | **Moderate**  Due to serious risk of bias, Due to serious imprecision^10^ | Compared with other immunosuppressants, telitacicept+ other immunosuppressants may not reduce 24hUTP. |
|  |  | Difference: **MD 0.05 lower**  (CI 95% 0.41 lower - 0.32 higher) | |  |  |
| eGFR - Telitacicept VS Other immunosuppressants | Measured by:  Scale: -  Based on data from 318 participants in 6 studies^11^ |  |  | **Moderate**  Due to serious risk of bias^12^ | Compared with Other immunosuppressants, telitacicept may not reduce eGFR. |
|  |  | Difference: **MD 3.44 higher**  (CI 95% 2.61 lower - 9.49 higher) | |  |  |
| eGFR - Telitacicept+ other immunosuppressants VS Other immunosuppressants | Measured by:  Scale: -  Based on data from 135 participants in 2 studies^13^ |  |  | **Moderate**  Due to serious risk of bias^14^ | Compared with other immunosuppressants, telitacicept+ other immunosuppressants may not reduce eGFR. |
|  |  | Difference: **MD 7.61 higher**  (CI 95% 2.10 lower - 17.31 higher) | |  |  |
| Scr - Telitacicept VS Other immunosuppressants | Measured by:  Scale: -  Based on data from 124 participants in 3 studies^15^ |  |  | **Very low**  Due to serious risk of bias, Due to very serious imprecision^16^ | Compared with Other immunosuppressants, we are uncertain whether telitacicept increases or decreases Scr. |
|  |  | Difference: **MD 2.12 higher**  (CI 95% 18.55 lower - 22.79 higher) | |  |  |
| Scr - Telitacicept+ other immunosuppressants VS Other immunosuppressants | Measured by:  Scale: -  Based on data from 58 participants in 1 studies^17^ |  |  | **Very low**  Due to serious risk of bias, Due to very serious imprecision, Due to serious risk of bias, Due to very serious imprecision^18^ | Compared with Other immunosuppressants, we are uncertain whether telitacicept+ other immunosuppressants increases or decreases Scr. |
|  |  | Difference: **MD 13.0 lower**  (CI 95% 33.79 lower - 7.79 higher) | |  |  |
| Alb - Telitacicept VS Other immunosuppressants | Measured by:  Scale: -  Based on data from 119 participants in 2 studies^19^ |  |  | **Very low**  Due to serious risk of bias, Due to very serious imprecision^20^ | Compared with other immunosuppressants, we are uncertain whether telitacicept increases or decreases Alb. |
|  |  | Difference: **MD 1.48 higher**  (CI 95% 0.08 lower - 3.04 higher) | |  |  |
| Alb - Telitacicept+ other immunosuppressants VS Other immunosuppressants | Measured by:  Scale: -  Based on data from 135 participants in 2 studies^21^ |  |  | **Very low**  Due to serious risk of bias, Due to very serious imprecision^22^ | Compared with other immunosuppressants, we are uncertain whether telitacicept+ other immunosuppressants increases or decreases Alb. |
|  |  | Difference: **MD 1.5 higher**  (CI 95% 0.61 lower - 3.61 higher) | |  |  |

1. Systematic review [2] with included studies: Tianjun Zhang 2025, Meng Wang 2024, Yangyang He 2025 **Baseline/comparator** Control arm of reference used for intervention .
2. **Risk of Bias: serious. Imprecision: serious.**
3. Systematic review [2] with included studies: Jing Luo 2024, Fanqian Cheng 2025 **Baseline/comparator** Control arm of reference used for intervention .
4. **Risk of Bias: serious.** due to [observational study]; **Inconsistency: serious.** due to [Point estimates vary widely]; **Imprecision: serious.** Wide confidence intervals and data from one study.
5. Systematic review [2] with included studies: Lingqiu Dong2025, Yangyang He 2025, Meng Wang 2024, Tianjun Zhang 2025 **Baseline/comparator** Control arm of reference used for intervention .
6. **Risk of Bias: serious.** due to [reason]; **Imprecision: serious.**
7. Systematic review [2] with included studies: Meng Wang 2024, Tianjun Zhang 2025, Yangyang He 2025, Hui Li 2025, Lingqiu Dong2025, Kun Yang 2025 **Baseline/comparator** Control arm of reference used for intervention .
8. **Risk of Bias: serious.**
9. Systematic review [2] with included studies: Jing Luo 2024, Fanqian Cheng 2025 **Baseline/comparator** Control arm of reference used for intervention .
10. **Risk of Bias: serious. Imprecision: serious.**
11. Systematic review [2] with included studies: Meng Wang 2024, Tianjun Zhang 2025, Hui Li 2025, Yangyang He 2025, Lingqiu Dong2025, Kun Yang 2025 **Baseline/comparator** Control arm of reference used for intervention .
12. **Risk of Bias: serious.**
13. Systematic review [2] with included studies: Fanqian Cheng 2025, Jing Luo 2024 **Baseline/comparator** Control arm of reference used for intervention .
14. **Risk of Bias: serious.**
15. Systematic review [2] with included studies: Hui Li 2025, Kun Yang 2025, Tianjun Zhang 2025 **Baseline/comparator** Control arm of reference used for intervention .
16. **Risk of Bias: serious.** due to [reason]; **Imprecision: very serious.**
17. Systematic review [2] with included studies: Jing Luo 2024 **Baseline/comparator** Control arm of reference used for intervention .
18. **Risk of Bias: serious. Imprecision: very serious.**
19. Systematic review [2] with included studies: Meng Wang 2024, Tianjun Zhang 2025 **Baseline/comparator** Control arm of reference used for intervention .
20. **Risk of Bias: serious. Imprecision: very serious.**
21. Systematic review [2] with included studies: Jing Luo 2024, Fanqian Cheng 2025 **Baseline/comparator** Control arm of reference used for intervention .
22. **Risk of Bias: serious. Imprecision: very serious.**

**Figure S1-S6 The results of sensitivity analysis**

Figure S1. The sensitivity analysis result of CR

Figure S2 The sensitivity analysis result of 24hUPQ

Figure S3 The sensitivity analysis result of eGFR

Figure S4 The sensitivity analysis result of Scr

Figure S5 The sensitivity analysis result of Alb

Figure S6 The sensitivity analysis result of ADRs
